# Land surface black-sky albedo at a fixed solar zenith angle and its relation to forest structure during peak growing season based on remote sensing data

**DOI:** 10.1016/j.dib.2020.105720

**Published:** 2020-05-20

**Authors:** Sara Alibakhshi, Thomas W. Crowther, Babak Naimi

**Affiliations:** a Department of Built Environment, School of Engineering, Aalto University, P.O. Box 14100, 00076 Aalto, Finland; b Institute of Integrative Biology, ETH Zürich, Universitätstrasse 16, P.O. Box 8092, Zürich, Switzerland; c Department of Geosciences and Geography, University of Helsinki, P.O. Box 64, 00014 Helsinki, Finland

**Keywords:** albedo, forest structure forest density, tree cover, leaf area index

## Abstract

Satellite data provide the opportunity to explore different land surface properties, such as albedo (reflectivity) and forest structure, for multidisciplinary purposes. We estimated land surface black-sky albedo at shortwave, near-infrared and visible spectral regions at a fixed solar zenith angle (i.e., 38^∘^) during peak growing season in 2005 on a global scale. In addition, we estimated the links between albedo and forest structure variables including forest density [the number of trees/km^2^], tree cover [percent], and leaf area index [m^2^/m^2^] over pure forest pixels during peak growing season in 2005 on a global scale. We acquired and processed remotely sensed variables from moderate resolution imaging spectroradiometer (MODIS) and Landsat satellite images. This article provides 1) dataset of black-sky albedo at fixed solar zenith angle at a 1-km spatial resolution, 2) comparison between black-sky albedos at fixed solar zenith angle and local noon at a 1-km spatial resolution that are grouped based on forest types with the classes of evergreen needleleaf, evergreen broadleaf, deciduous needleleaf, deciduous broadleaf, mixed and woody savannah forests, and also the major biome zones including boreal, mediterranean, temperate and tropical region. 3) the links between black-sky albedo at fixed solar zenith angle and forest structure using generalized additive models at a 0.5-degree spatial resolution during peak growing season in 2005. The pre-processing steps to enhance the accuracy of these datasets include: (1) identifying pure forest pixels, (2) excluding high slope pixels and those covered partially by water in the albedo product using high spatial resolution water (i.e., 30-m spatial resolution) and slope (i.e., 90-m spatial resolution) masks, and (3) using the most recent collection (collection 6) of MODIS satellite images. More details and interpretations of these datasets can be found in Alibakhshi et al. (2020) [1].

Specifications table

**Table:** 

Subject	Global and Planetary Change
Specific subject area	Remote sensing of forests
Type of data	Spatial raster layers (GeoTiff format)
How data were acquired	We used Google Earth Engine to obtain/estimate albedo [2], [3], [4], forest types, forest structure variables including forest density [the number of trees/km^2^] [5], tree cover [percent] [6], leaf area index [m^2^/m^2^] [7,8], forest type [9], ecoregion map [10], slope [11], and water body [12] raster layers. All statistical analyses and visualisations were performed in R statistical software [13], QGIS [14], and Google Earth Engine [15]. We used different R packages, including “raster” [16], “rts” [17], “mgcv” [18], “phenopix” [19], “usdm” [20], “ggplot2” [21], and “rasterVis” [22].
Data format	Raw
	Filtered
	Analysed
Parameters for data collection	Albedo, forest structure, and ancillary data were obtained for the year ∼2005. All the data are aggregated at a 1-km spatial resolution and are analysed using a geographic coordinate system. The map of the links between albedo and forest structure is provided at a 0.5-degree spatial resolution.
Description of data collection	Google Earth Engine was used to obtain and process the data. Part of data processing was conducted in the R software.
Data source location	Global
Data accessibility	https://data.mendeley.com/datasets/54t5cgt5yy/1
Related research article	1. Alibakhshi, S.; Naimi, B.; Hovi, A.; Crowther, T. W.; Rautiainen, M. Quantitative analysis of the links between forest structure and land surface albedo on a global scale. *Remote Sens. Environ.* 2020 (*Accepted*).

## Value of the data

The data of albedo can be used:

○ to explore the surface energy balance system and to understand the underlying mechanisms, since albedo is a critical factor in energy balance modelling.
○ to understand the role of forest structure in modulating albedo that has potential applications, e.g., in forest management by evaluating the optimal geographical locations to modulate albedo;
○ to assess the difference between albedo at local noon and albedo at fixed solar zenith angle (i.e., 38^∘^) during peak growing season;
○ to provide empirical evidence of the links between albedo and forest structure for modelers in validation purposes, etc.
○ for making land-use decisions and further detailed analysis requires the magnitude of the links between albedo and forest structure.
○ in different fields of studies such as land surface modeling, carbon cycle modelling, forestry, economy, biodiversity conservations and climate studies to enhance the available knowledge.

## 1 Data

This article provide the raster layers of black-sky albedo at a fixed solar zenith angle (i.e., 38^∘^) in shortwave (SW), near-infrared (NIR) and visible (VIS) spectral regions, as well as the difference between black-sky albedo at local noon and black-sky albedo at the fixed solar zenith angle with a 1-km spatial resolution during peak growing season in 2005 on a global scale. In addition, the article provided the map of the links between forest structure and albedo at a 0.5-degree spatial resolution during the peak growing season in 2005 on a global scale [1]. All the data of this file use a geographic coordinate system. We only focused on pure forest pixels, meaning that when we aggregated the forest type map from 500-m to 1-km using a modal function [9], if all the 500- resolution pixels had the same forest type within a 1-km resolution pixel, we considered as a pure pixel. We classified impure pixels as mixed or woody savanna forests based on tree cover [1]. We provided all the datasets in a zip folder called “albedo_PGS.zip”. This file includes, 1) Raw data of SW, NIR and VIS black-sky albedo at a fixed solar zenith angle; 2) Raster layers of the relationship between black-sky albedo and each forest structure variable separately under name of, for example, spatial_gam_nir_density.tif that means links between near-infrared albedo and forest density; 3), and the relationship between albedo and all forest structure variables under name of, for example, spatial_gam_nir_all.tif that, means links between "nir albedo" and "forest density, tree cover, and leaf area index". The delta_SW_albedo.zip file includes the raster layers of delta albedo i.e., at fixed SZA albedo of 38^∘^ – local solar noon albedo). In each file, “br” means boreal region, “me” means mediterranean region, “tem” means temperate region, “tr” means tropical region. The values after br, me, tem and tr, including one to five and eight represent evergreen needleleaf, evergreen broadleaf, deciduous needleleaf, deciduous broadleaf, mixed and woody savannah forests, respectively.

## 2 Experimental design, materials, and methods

### 2.1 Overview

The standard albedo product of MODIS (MCD43A3) provides albedo for each pixel at the local solar noon, meaning that different solar zenith angles (SZAs) can be observed at different geographical locations [3]. However, land surface albedo values are strongly influenced by SZA variations. Therefore, albedo values can be different under different SZAs in the same forest structure and atmospheric condition. Hence, eliminating the effects of SZA variations on albedo is crucial to isolate the influence of varying factors to only forest structure to explore the links between albedo and forest structure. We prepared the albedo at a fixed solar zenith angle and local noon and estimated how they can be different over pure pixels in peak growing season.

In this file, we prepared datasets in several steps. First, we downloaded albedo [2,4], tree cover [6], forest density [5] as well as time series of leaf area index (LAI) [23,24] satellite images for the year ∼2005. We used high-quality LAI to determine the timing of peak growing season at each sub-biome, noting that the peak growing season can happen at different times over a year across different geographical locations [1]. Then, we created a dataset of albedo and forest structure based on the timing of the peak growing season. Next, we performed a set of pre-processing steps on the datasets used in this file to keep only high-quality pixels. Following this, we estimated back-sky albedo at the fixed solar zenith angle using the model parameter dataset of bidirectional reflectance distribution function (BRDF) and SZA. Then, we obtained black-sky albedo at local noon from MCD43A3 product. Finally, we explored the differences between albedo at fixed SZA and local noon that lead us to use albedo at fixed solar zenith angle to produce the map of the links between forest structure and albedo using generalized additive models (GAMs) at peak growing season [25,26].

### 2.2 Black-sky albedo at local noon

We prepared the dataset of black-sky albedo at local noon using daily albedo of MODIS satellite images (MCD43A3) with a 500-m spatial resolution during peak growing season in 2005 [3] (Fig. 1 of this file). Black-sky albedo describes the albedo under direct illumination conditions (i.e., the sun as the point source of illumination). We used two MODIS products, MCD43A3 and MCD43A2, containing daily BRDF/albedo and their quality values at 500-m spatial resolution. Using the quality values, we kept only good-quality pixels (full BRDF inversions). Currently, MCD43A2 has no topography-related quality information, and it may result in some errors in forests located in rugged terrains [27]. To avoid this error, we excluded pixels with a topographic slope greater than 10°. Furthermore, we avoided the effects of very low reflectance of water on albedo values by using a high-resolution water mask with 30-m resolution [12] and excluding the pixels with water that covered greater than 5% of a pixel area.

**Fig. 1a:**
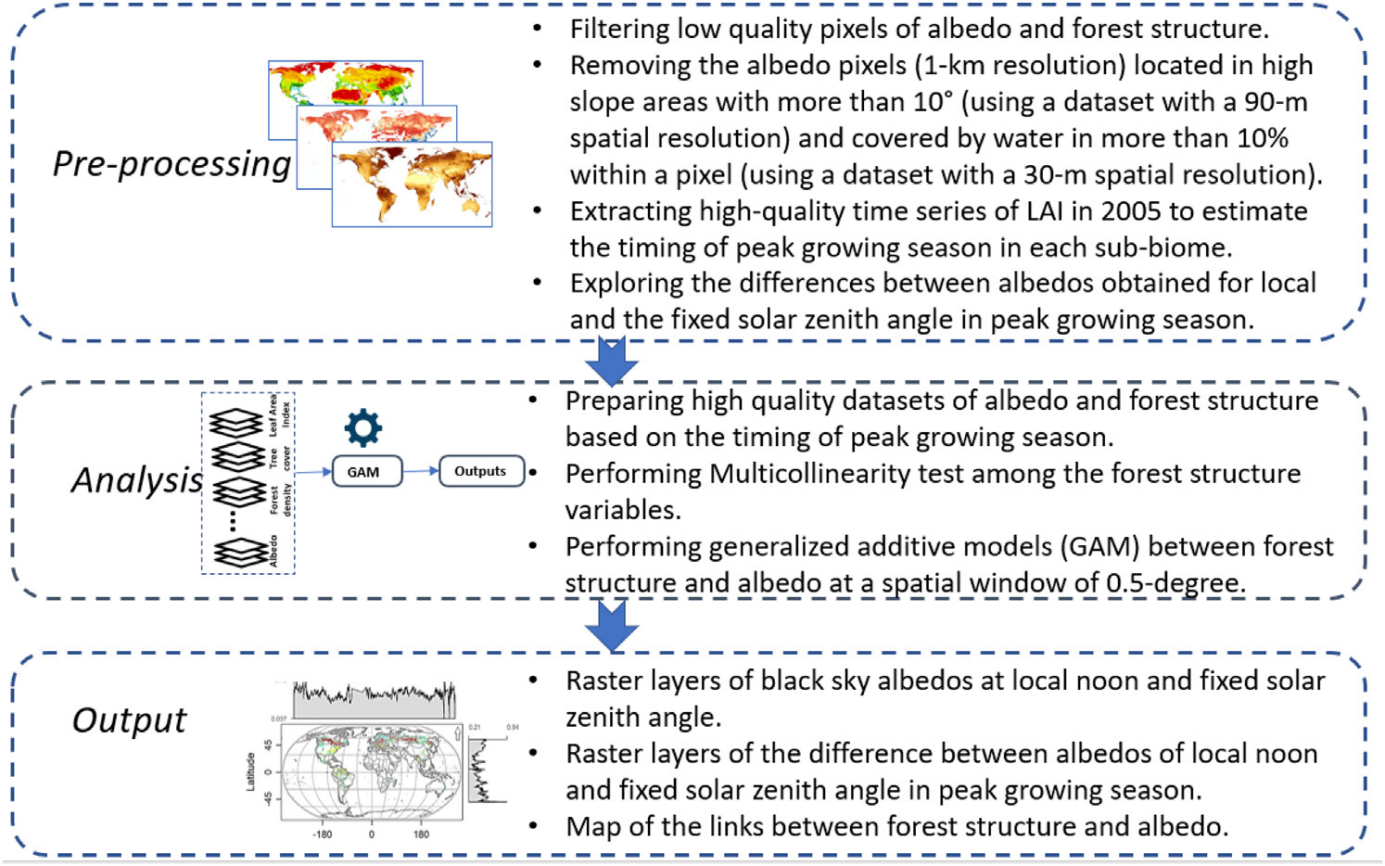
The flowchart of steps to prepare the datasets in this article.

**Fig. 1:**
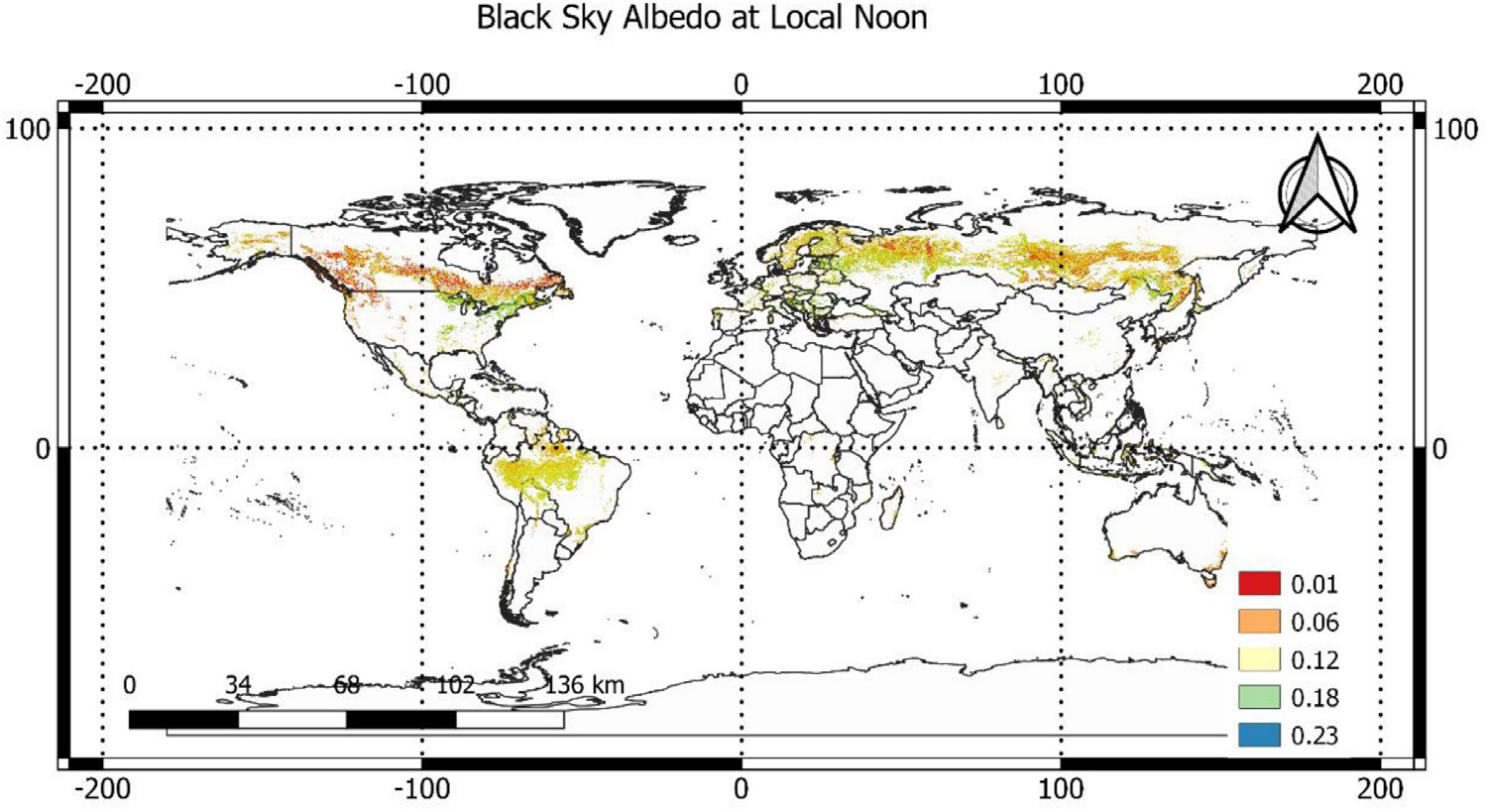
Spatial distribution of SW black-sky albedo at local noon with a 1-km resolution during the peak growing season in the year 2005.

### 2.3 Albedo at fixed solar zenith angle

**Fig. 2:**
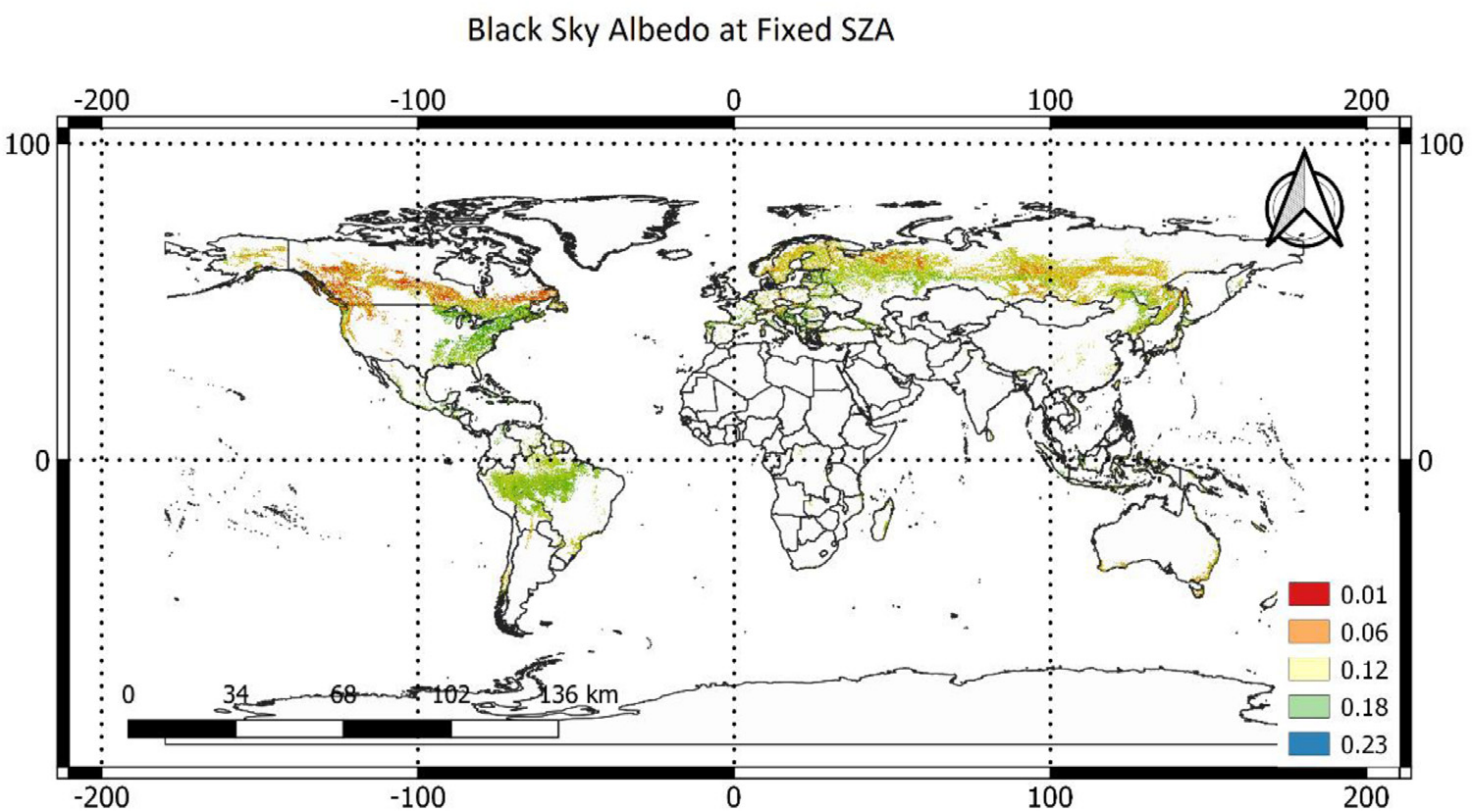
Spatial distribution of SW black-sky albedo at a SZA of 38^∘^ with a 1-km resolution during the peak growing season in the year 2005.

To estimate black-sky albedo at fixed SZA, we obtained the model parameter dataset of BRDF and SZA using MCD43A1, and MCD43A2 products of MODIS satellite images using the formula in Strahler et al., (1999) (Fig. 2 of this file) [28]. For more information on the estimation of albedo at a fixed solar zenith angle, please see Alibakhshi et al. (2020) [1]. In general, albedo values range between 0 and 1 and forest albedo in this dataset has a value of less than 0.4 over pure forest pixels during peak growing season.

### 2.4 Comparison between black-sky albedo at local noon and fixed SZA

**Fig. 3:**
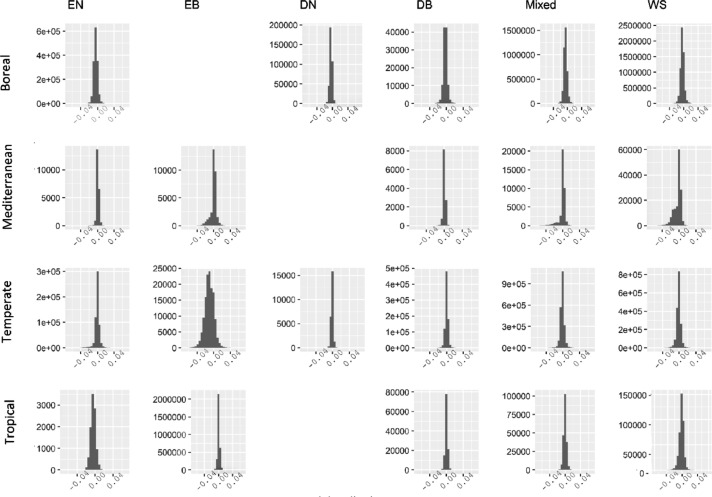
Delta albedo (i.e., fixed SZA albedo – local solar noon albedo) at SZA of 28^∘^. The rows refer to the major biome zones and columns refer to forest type, including evergreen needleleaf (EN), evergreen broadleaf (EB), deciduous needleleaf (DN), deciduous broadleaf (DB), mixed forests (Mixed), and woody savannah forests (WS). In each graph, the x-axis refers to the delta albedo, and the y-axis refers to the number of pixels.

The mean of SZA for all the forest pixels during the peak growing season in 2005 was 28^∘^ [1]. However, 28° would be an unrealistic value for the boreal region. During the peak growing season in 2005, the mean SZA of the boreal region was 38°. Therefore, we prepared the dataset of the difference between the two albedo values (i.e., fixed SZA albedo – local solar noon albedo) which hereafter is called delta albedo for both SZA of 28° and 38° (Fig. 3:4 and Table 1 of this file). The delta albedo datasets have a range of values between -0.05 to 0.05 in both SZA of 28° and 38° (Fig. 3:4).

**Fig. 4:**
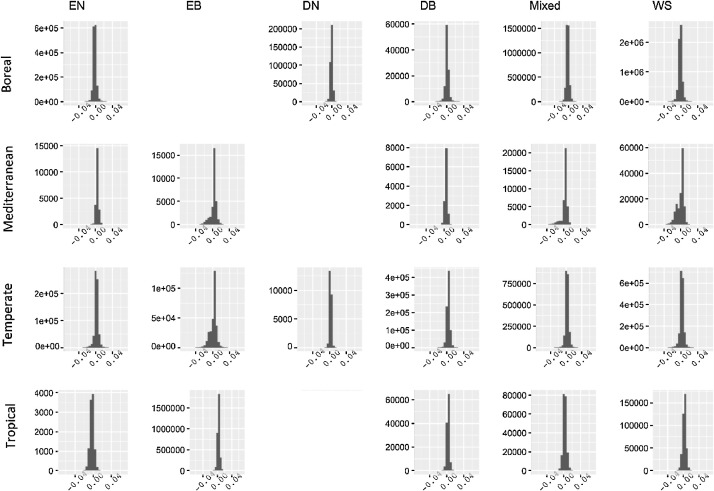
Delta albedo (i.e., fixed SZA albedo – local solar noon albedo) at SZA of 38^∘^. The rows refer to the major biome zones and columns refer to forest type, including evergreen needleleaf (EN), evergreen broadleaf (EB), deciduous needleleaf (DN), deciduous broadleaf (DB), mixed forests (Mixed), and woody savannah forests (WS). In each graph, the x-axis refers to the delta albedo, and the y-axis refers to the number of pixels.

**Table 1:** Delta albedo (i.e., fixed SZA albedo – local solar noon albedo) during peak growing season in 2005. The columns refer to forest types including evergreen needleleaf (EN), evergreen broadleaf (EB), deciduous needleleaf (DN), deciduous broadleaf (DB), mixed forests (Mixed), and woody savannah forests (WS).

	EN	EB	DN	DB	Mixed	WS
Boreal	0.002137	NA	0.001074	-0.00089	0.002255	0.001675
Mediterranean	-0.00441	-0.00309	NA	-0.00402	-0.00208	0.00283
Temperate	-0.00193	-0.00181	-0.0019	-0.00366	-0.00247	-0.00206
Tropical	0.002504	-0.00355	NA	-0.00338	-0.00265	-0.00297

### 2.5 Analysis to explore the links between albedo and forest structure

We provided the dataset for the relationships between forest structure and albedo that are explored in Alibakhshi et al. (2020) [1]. The main procedure includes: fitting a set of GAMs over a globally extended coarse resolution pixels (50 km × 50 km) [20], where each coarse pixel was used as a spatial unit over which we fitted a GAM. In each GAM, albedo was a response variable and forest structure was explanatory variable. Then, we assigned the model's R^2^ to the coarse grid cell. In the dataset of the links between forest structure and albedo, we reported the R^2^ of the relationship pixel-wise that range between 0.12 and 0.85. In this dataset, 0.12 refers to a weak relationship between forest structure and albedo, and 0.85 refers to a strong relationship.

## Declaration of Competing Interest

None
